# Is there room for mothers' agency in the choice to breastfeed? A qualitative analysis of mothers’ views on messages promoting breastfeeding in Quebec

**DOI:** 10.18332/ejm/174931

**Published:** 2024-01-08

**Authors:** Thomas Delawarde-Saïas, Coralie Mercerat, Marion Adamiste, Émilie Pigeon-Gagné, Cécile Delawarde, Johanna Nouchi, Janie Comtois, Sarrah Bakhty, Julie Poissant

**Affiliations:** 1 University of Quebec in Montreal, Montreal, Canada; 2 TELUQ University, Quebec, Canada; 3 Laval University, Quebec, Canada; 4 Centre hospitalier de l'Universite de Montreal, Montreal, Canada

**Keywords:** breastfeeding, maternity, public health messages, professionals’ discourse

## Abstract

**INTRODUCTION:**

This exploratory cross-sectional study focuses on the experiences of mothers regarding health messages promoting breastfeeding. The objective is to describe the content and context in which messages are conveyed.

**METHODS:**

A total of 944 new mothers responded to a questionnaire (15–31 January 2021) on their perception of health messages promoting breastfeeding and their feeling of agreement towards these messages, their intention to breastfeed, incentives received, and their relationship with the professionals. Frequencies were carried out for all non-textual data and textual data were analyzed using content thematic analysis. The recruitment was made through social media and snowball effect.

**RESULTS:**

Most of the respondents reported wanting to breastfeed; 91% breastfed their child, 80.8% participants agreed with the messages they received, and 67.9% of respondents strongly agreeing that breastfeeding was the best choice for their child. Moreover, the content of the messages could sometimes be judgmental and coercive, leading to emotions such as guilt. Sixty-two women also reported a lack of support when they expressed their desire or their need to feed their baby in other ways (e.g. breastmilk with bottles or formulas).

**CONCLUSIONS:**

The perceived issue of breastfeeding messages was not the content itself, but the way in which information was conveyed. Failure to take mothers' difficulties into account and failure to present alternatives to breastfeeding were seen as major issues by women. This study highlights the importance of rethinking the way in which information is provided by professionals, in order to reinforce the autonomy of new mothers regarding the feeding of their child.

## INTRODUCTION

Breastfeeding is recognized as an optimal feeding method for babies. It is reported to decrease infant mortality^1^, to protect babies against certain infectious diseases, food allergies, eczema, and asthma^2^, and also to promote the child’s cognitive abilities^3^. For mothers, breastfeeding is reported to be a protective factor for breast and ovarian cancers^4^, hypertension, and diabetes^5^. The World Health Organization (WHO), United Nations International Children’s Emergency Fund (UNICEF), the Public Health Agency of Canada and Health Canada (Government of Canada) actively recommend exclusive breastfeeding for the first six months of a child’s life. To promote breastfeeding, the WHO and UNICEF developed the Baby-Friendly Initiative (BFHI) in the 1990s. This initiative is an international program based on the practical application of recommendations to protect and support breastfeeding. In Canada, there were 155 baby-friendly facilities in 2018, 107 of which were in the province of Quebec^6^. Moreover, Quebec has developed an active policy in favor of breastfeeding through its National perinatal policy^7^.

According to Miracle and Fredland^8^, regardless of the personal beliefs held by healthcare professionals, they have an ethical responsibility to encourage breastfeeding. Their ability to support and encourage mothers has been shown to be related to the choice to breastfeed and the duration of breastfeeding^9,10^. Mothers who are encouraged to breastfeed are four times more likely to breastfeed than those who were not encouraged^11^. Also, mothers whose caregivers have neutral or negative attitudes towards breastfeeding and who are not pressuring towards it, are reported to stop breastfeeding prematurely^9,11^. Thus, there is an influence of professionals’ attitudes, behaviors, and discourse on mothers’ choices and behaviors. Several studies confirm that professionals are aware of the influence they can have on mothers, particularly in their choice to breastfeed or not^12^. As a result, these individuals hold some power as experts and are perceived as authority figures^13^.

After more than two decades of intensive promotion, women who are still unaware that breastfeeding promotes their babies’ optimal development are rare^14^. It would be almost impossible to miss the advertisements and discourses promoting breastfeeding that suggest that ‘breastfeeding is the only ethically acceptable option in infant feeding’^15^. But beyond the informative aspects, focused on health promotion, a real pressure seems to be placed on women to submit to the social norm of breastfeeding^15-18^. In fact, the Quebec Ministry for Health and Social Services’ website states that the ‘(Baby-Friendly) Initiative aims to create care settings where breastfeeding is the norm’^19^. Thus, choosing not to breastfeed represents a deviation from this norm.

The ‘breast is best’ argument is often reported by mothers as having a ‘prescriptive’, ‘unhelpful’^20^, and moralistic dimension^21^. The attitude and discourse of health professionals was described as ‘pushy’ in the sense that they pressure mothers to breastfeed^22^ and criticize them when they do not. Some women described professionals as giving standardized, insensitive, and judgmental advice^23^. According to Bétrémieux^24^, there is, therefore, an urgent need to rethink the professionals’ discourse and that ‘the will to inform [for the professionals] should replace the will to convince’^24,25^.

This pressure is often described as unreasonable by mothers, dispossessing them of their agency, and influencing their emotional well-being, thus generating negative emotions^26,27^ that may impact their own well-being, as well as their babies’^28,29^. Pressure on mothers is reportedly associated with increased rates of postpartum depression^29^. They then substitute this needs assessment with recommendations that may be detached and irrelevant. These practices, when they are not respectful of women’s choices match the WHO description of violence against women (‘gender-based violence that results in physical, sexual or mental harm or suffering to women’). The quality of care provided to women and the abuse they may experience in a medical, but particularly obstetrical context, have been the focus of attention in recent years^30^.

As part of a research program aimed at optimizing relationships between perinatal professionals and families, our team conducted a study to describe the experiences of women exposed to messages that promote breastfeeding. Our hypothesis was that, regardless of their intention or commitment to breastfeeding, they would receive messages promoting it. Some of these messages would have negative effects on their perinatal experience of motherhood, such as a sense of loss of agency.

The objective of this study is to describe the messages – the content and the context in which they are conveyed – perceived as congruent with mothers’ expectations and those perceived as inconsistent. This exploratory study focuses on the experiences of new mothers during birth preparation sessions, in the birth unit, and during the first weeks of their child’s life. We paid particular attention to negative experiences and those related to the loss of agency, in order to better highlight the areas of opportunity to be addressed with the caregivers and, ultimately, to prevent forms of gender-based violence and gender inequalities.

## METHODS

### Participants and procedure

This cross-sectional study was conducted using a sample recruited online. It received the approval of the ethics committee of the Université du Québec à Montréal (CIEREH #2021-3521). We sought participation from individuals who had had a child within the past two years (the only inclusion criterion), regardless of other sociodemographic characteristics or number of other motherhood experiences. The population was recruited via ads posted on social networks, for example Facebook groups of new parents, who could transfer the survey link to their network (snowball recruitment). Sampling bias was tolerated by the research team as this mixed-methods study aimed to describe the different forms of breastfeeding promotion messages received. Thus, the analyses were not intended to be representative of the population, but of the different types of messages produced by professionals. The contact message was the following (in French): ‘Are you the mother of a child under the age of two? We would like to know about your experience with health professionals regarding your choice of food for your baby’.

### Instruments

Participants could then access a questionnaire hosted on SurveyMonkey^®^ addressing: 1) the experience of having been encouraged to breastfeed; 2) the nature of the messages received and the feeling of agreement with these messages (on an agreement scale from 0=strongly disagree to 10=strongly agree); 3) the intention to breastfeed and the final mean chosen to feed their child; and 4) for women who had fed their child with commercial formulas, their relationship with the professionals regarding this decision. All of these sections included open-ended questions, allowing participants to recount both good and bad experiences in as much detail as they wished.

### Analysis

Flat sorts (frequencies) were carried out for all the non-textual data. The qualitative (textual) data were analyzed using Braun and Clarke’s^31^ content thematic analysis method. This method makes it possible to group together sequences of text relating to the same concepts. The categorization process is carried out through a six-step process (familiarization with the corpus, code generation, theme generation, theme refinement, theme definition and data reporting). This process was carried out by one of the research team members, after an initial coding phase with a second team member, in order to discuss disagreements towards the coding, to finalize the coding grid, and to ensure the relevance of the coding.

## RESULTS

A total of 944 participants meeting the recruitment criteria participated in the research. Of these, 94% intended to breastfeed during their pregnancy and 91% actually breastfed their infants at birth, either with breast milk or a bottle of breast milk. Results show that 54% (n=510) of the participants reported having received one or more breastfeeding prompts from health professionals. Of these, 19.2% reported ‘being neutral’ or ‘disagreeing’ with the messages received (score of ≤5 on the agreement scale).

The results presented here were categorized according to whether participants reported ‘agreeing with the content of the messages’ (6–10 on the agreement scale) or ‘neutral/disagreeing’ (<6 on the agreement scale).

### Positively received messages (agreement toward the messages)

Of the 510 participants who reported receiving messages in support of breastfeeding, 80.8% said they agreed with these messages; 12.9% somewhat agreed with the messages (6 or 7 on the agreement scale) and 67.9% strongly agreed (8–10 on the agreement scale) with the messages. These pro-breastfeeding messages addressed:

- The opportunity offered to women to receive the message, as stated by a participant:

*‘First they asked me if I thought about breastfeeding, when I said yes they gave me all the information.’* (Participant 85)

- The importance of breastfeeding, including: 1) The quality of breast milk regarding an inherent quality:

*‘Breastfeeding strengthens the bond between mother and child as well as the child’s self-esteem because it makes him/her feel secure.’* (Participant 627)

*‘Breastfeeding is better for the baby because it gives him/her antibodies and he/she will rarely get sick.’* (Participant 232)

*‘It reduces the risk of sudden death.’* (Participant 477)

*‘Breast milk offers much more nutrients than formula.’* (Participants 238 and 1110)

and a relative quality (compared to commercial formulas) with comments such as: *‘Breastfeeding is the best for the baby.’*; 2) Convenience of breastfeeding with words such as *‘convenient’*, *‘always ready’*, *‘economical’*; 3) The ease of this natural feeding method, as reported by one of the participants:

*‘[I was told that] I have good breasts to breastfeed and it will be easier for baby and me; it is natural and “it will come”; [it]doesn’t hurt.’* (Participant 1101)

and 4) Breastfeeding as a ‘gift’ to the baby as a woman reported having received the following message:

*‘You know your baby will thank you for breastfeeding.’* (Participant 505)

- The support offered to women making this choice, i.e. exclusive breastfeeding, as stated by a participant:

*‘I already wanted to breastfeed. I received encouragement from my midwives and support and technical help. They congratulated me several times for choosing to invest 100% in exclusive breastfeeding … Interesting stuff that helped me understand what is happening physiologically in my body and for baby.’* (Participant 937)

However, many respondents shared messages involving pressure or judgment, as reported by some women of the sample, for example:

*‘I agree that the message should be passed on but I feel uncomfortable vs the pressure it puts on mothers who would choose not to breastfeed or who just can’t.’*

*‘You have to breastfeed, no other way is good for your child.’*

*‘It is recommended by the WHO until 2 years of age’*.

Finally, several respondents mentioned pressuring messages from healthcare providers related to the difficulties encountered:

*‘You have to breastfeed even if it hurts too much.’*

*‘All women can breastfeed, no matter what difficulties you go through with help, you can always continue to breastfeed. You have to try and persist.’*

### Negatively received messages (disagreement toward the messages)

Ninety-seven respondents (19.2%) indicated that they somewhat disagreed with the messages they received (0–5 on the agreement scale) regarding breastfeeding. This part of the results focuses on the message itself, allowing for a better understanding of the negative experiences and constituting most of the testimonies of mothers who did not agree with the messages received.

The disagreement could be related to the content of the message. The main themes of these messages are presented as follows. Doubt about the content concerned the lack of scientific support for the message, the lack of information (incomplete message) or the erroneous nature of the information shared:

*‘I did not disagree. I just don’t know if the information regarding nutritional values is true.’* (Participant 139)

Some respondents also mentioned that the message could be restrictive, emphasizing the mother’s personal choice to breastfeed and the importance of presenting alternatives without judgment (some information was sometimes withheld to convince people of the benefits of breastfeeding):

*‘I would have preferred that they asked me what my choice was before giving me more information and especially not insinuating that other options were not valid.’* (Participant 189)

Some respondents also mentioned that the mother’s mental health was as important as the nutritional benefits in the breastfeeding process. Then, some mothers reported that the postpartum difficulties they experienced were not adequately addressed. Finally, many women argued that the emphasis was not on the right message. While the message focused on the health and developmental benefits of breastfeeding, the message did not consider the mother’s discomfort with breastfeeding, nor her needs:

*‘The message never takes the mother’s needs into account, both physical and psychological. I never got any advice or information about the other option. I felt it was an obligation as a new mother and woman.’* (Participant 77)

The women also reported comments related to the way the message was delivered to them. Some reported that the message was guilt-inducing, that they felt like bad mothers, and that they were being infantilized by professionals:

*‘I personally had a lot of trouble breastfeeding and my baby was constantly hungry. I was still pushed to continue by playing on my guilt about feeding my child well. No health care professional offered to switch to bottle feeding when my baby was losing weight and I was not doing well after the birth. We made the decision as a family to switch to bottle feeding and had no support or information from the nurses we consulted.’* (Participant 60)

Some participants also reported that the medical staff were relentless in their efforts to breastfeed and some even reported being physically forced to do so:

*‘Had I not been so exhausted I would have just refused to be touched – it wasn’t working and they were hurting me and, it surely wasn’t any more comfortable for my child.’* (Participant 128)

### Support or lack of support in the decision not to exclusively breastfeed

Sixty-two women indicated that they did not feel supported when they had finally decided to bottle-feed. They reported four types of reactions from professionals:

- Lack of reaction; some women reported that once the baby regained his/her birth weight, community health services told them that they could no longer support them:

*‘When the baby regained his birth weight, I was told that the [proximity health center] no longer offered support and that I had to refer to the pediatrician or a breastfeeding support.’* (Participant 1274)

- No alternative information to breastfeeding; a woman reported her experience as followed:

*‘In prenatal classes, during the breastfeeding session, when I asked her if she was going to talk about formulas, she answered: “You can find everything on the internet.”.’* (Participant 77)

- Pressure, dissuasion, judgment; some women reported feeling a pressure to breastfeed and some tension from the professionals, as stated by one of the mothers:

*‘I felt the nurses were more stressed than I was that I was missing my breastfeeding because I wasn’t able to breastfeed my baby boy and get him to drink from my breast.’* (Participant 1062)

- Forced breastfeeding; one woman in our sample reported being discouraged from expressing milk, receiving messages from professionals that she was selfish for not wanting to breastfeed exclusively:

*‘Refusal of the nurses and doctor on site [in the hospital] to let me express my milk with my own pump and refusal to give me a bottle of formula since I could not express my milk … I wanted to breastfeed only once in a while and pump my milk regularly. They did everything to discourage me even though it’s the same milk … it seems that it’s selfish to not want to be the only one who can feed baby.’* (Participant 954)

One hundred and forty women mentioned, however, that they had received some form of support in their choice not to breastfeed exclusively (even though breastfeeding remained the preferred method of feeding according to the professionals encountered by these women). In this regard, women sometimes observed a discrepancy between the discourse of professionals who advised them:

*‘My obstetrician and my doula (birth attendant) reassured me that we needed a mother in good shape rather than breastfeeding at all costs. The postpartum nurses were a little more insistent but quickly saw the level of stress it put me under and agreed to provide formula.’* (Participant 682)

Despite what was mentioned above, regarding the support received, women reported that they were at times listened to in their choice. Sometimes the message conveyed was that ‘the important thing is that the baby is fed’, other times they felt they were respected in their choice because it was not their first pregnancy or because they had real difficulties in breastfeeding. At times, the bottle was suggested and even encouraged by the professionals, because the milk in the bottle was breast milk and not formula.

## DISCUSSION

The objective of this study was to describe the messages – content and context – perceived as congruent with mothers’ expectations and more specifically those perceived as inconsistent, in order to better understand the levers for women’s agency in deciding how to feed their newborns. The results show that a large majority (94%) of respondents wanted to breastfeed their child and that 91% breastfed them. Most women agreed with the messages they received about breastfeeding. Respondents reported receiving messages positively when professionals asked for their consent before giving them information or encouraged them in their choice to breastfeed exclusively.

However, some participants reported that the content of the messages could sometimes be judgmental and coercive to exclusively breastfeed, sometimes leading to guilt. Some of them also indicated that their discomfort (physical and psychological) was not considered in the messages conveyed by health professionals who – according to the respondents – did not take their wellbeing into account and did not suggest alternatives to breastfeeding. The difference between respondents who received the breastfeeding messages positively and those who received them negatively seems to be positioned around two central points: consent (to receive information), and choice of feeding method for their baby, referring directly to the notion of agency. As stated in other studies^32,33^, the promotion of exclusive breastfeeding is culturally embedded and influences professional practices and the latitude of decisions left to women. In this context, respondents reported that the information they received was one-sided in favor of exclusive breastfeeding, leaving them with a difficult choice to deviate from this ‘social norm’ and leading them to experience pressure. In our study, the pressure reported referred exclusively to health professionals, but Hvatum and Glavin^33^ also reported a form of pressure exerted by the women’s social network, reminding them of their duty to conform to the social norms.

In this regard, while some felt supported in their choice not to breastfeed (notably by bottle-feeding, but with breast milk), others mentioned non-supportive reactions from professionals, such as no reaction, no alternative information given, pressure or dissuasion. In its 2008–2018 perinatal policy (currently being renewed), the Government of Quebec reminds us that, while ‘breastfeeding is recognized as the best method of feeding infants’, it is important to ‘recall the importance of respecting the informed choice of the mother regarding breastfeeding’^9^. However, the results of our study indicate that the information about breastfeeding is strongly rooted in a hegemonic biomedical discourse, sometimes idealizing the psychosocial impact of breastfeeding by focusing only on mother–child attachment^34^. Therefore, the support is not always available or provided when women decide to slightly deviate from these medical guidelines – constituting the social normal – by not exclusively breastfeeding. Moreover, the mothers in this study stated at different times that they observed a discrepancy between the different health professionals they met with, some of whom informed or supported them in their choice, while others used more guilt-inducing discourses regarding their choice.

Finally, this study also emphasized that messages about breastfeeding, including (sometimes even physical) pressure on women to breastfeed, can also have psychological consequences for new mothers. Related to this result, several studies^30,32^ highlight that women can feel intrusion, distress, shame and humiliation, for example when health professionals manipulate their breasts to facilitate breastfeeding. It is also reported in the literature that mothers who make the choice not to breastfeed are more likely to experience guilt and shame than those who choose to breastfeed^35,36^.

### Strengths and limitations

This study contributes to the increase of current knowledge concerning the messages shared on breastfeeding. In fact, to our knowledge, it is the only study of its kind in the Quebec context. Thus, it lays the groundwork for future research on the messages conveyed to new mothers, whether on breastfeeding specifically or on other information related to the transition to parenthood, to avoid any form of gender-based inequalities or violence. Indeed, the results of this study highlight that women receive messages more positively when professionals respect their choice (to receive the information, to feed their child while breastfeeding or not), thus protecting the physical integrity and wellbeing of new mothers. From a perspective of social justice and accessibility to health services (in this case perinatal services), we hope that such results, congruent with existing literature from other countries, will be able to support the development of harmonious relations between users and health professionals, while respecting the autonomy and agency of new mothers.

However, this study also has some limitations. First, it is an exploratory study that interviewed women from different backgrounds. However, we had little control over the settings in which the survey was shared, as social media was used for the recruitment. We know that the questionnaire was widely shared in pro-breastfeeding leagues, which may have somewhat biased the results, especially since women may have found support for breastfeeding in these associations rather than from their healthcare professionals. Secondly, the survey method, although allowing us to approach a large number of people and being relevant to our current objective, has the limitation of not allowing to delve deeper into the experience of the participants. In this context, a future study deeply interviewing the mothers who had positive and negative experiences in relation to the messages received – for example, by using a convenience sample – could be relevant to deepen our understanding of this phenomenon and formulate possible solutions more clearly.

## CONCLUSIONS

As stated earlier, professionals have an ethical responsibility to offer breastfeeding as the preferred form of baby feeding. In this respect, the majority of women agree with this general principle. However, the results of this study, in congruence with other recent studies, highlight the importance of rethinking the way in which such information is provided, in order to reinforce the choice of new mothers regarding the feeding of their child. As the results of this study show, the issue is not the message (many respondents agreed that breastfeeding could be the best choice for their child), but rather the way in which such information is conveyed: failure to take mothers’ difficulties into account, and failure to present alternatives to breastfeeding. Finally, given the lack of knowledge about the negative effects of pressure to comply with breastfeeding, it is necessary to question the emphasis placed on the content of the message at the expense of the quality of the professional–mother interaction and consideration of mothers’ wishes.

## Data Availability

The data supporting this research are available from the authors on reasonable request.
